# Utility of Telomerase Gene Mutation Testing in Patients with Idiopathic Pulmonary Fibrosis in Routine Practice

**DOI:** 10.3390/cells11030372

**Published:** 2022-01-22

**Authors:** Julij Šelb, Katarina Osolnik, Izidor Kern, Peter Korošec, Matija Rijavec

**Affiliations:** 1 Department of Pathology, University Clinic of Respiratory and Allergic Diseases, 4204 Golnik, Slovenia; katarina.osolnik@klinika-golnik.si (K.O.); izidor.kern@klinika-golnik.si (I.K.); peter.korosec@klinika-golnik.si (P.K.); matija.rijavec@klinika-golnik.si (M.R.); 2 Medical Faculty, University of Ljubljana, 1000 Ljubljana, Slovenia; 3 Faculty of Pharmacy, University of Ljubljana, 1000 Ljubljana, Slovenia; 4 Biotechnical Faculty, University of Ljubljana, 1000 Ljubljana, Slovenia

**Keywords:** telomerase complex, idiopathic pulmonary fibrosis, genetic variants

## Abstract

Recent studies have suggested that causative variants in telomerase complex genes (TCGs) are present in around 10% of individuals with idiopathic pulmonary fibrosis (IPF) regardless of family history of the disease. However, the studies used a case-control rare variant enrichment study design which is not directly translatable to routine practice. To validate the prevalence results and to establish the individual level, routine clinical practice, and utility of those results we performed next generation sequencing of TCGs on a cohort of well-characterized consecutive individuals with IPF (diagnosis established according to ATS/ERS/JRS/ALAT guidelines). Of 27 IPF patients, three had a family history of idiopathic interstitial pneumonia (familial IPF) and 24 did not (sporadic IPF). Pathogenic/likely-pathogenic variants (according to American College of Medical Genetics criteria) in TCG were found in three individuals (11.1%) of the whole cohort; specifically, they were present in 2 out of 24 (8.3%) of the sporadic and in 1 out of 3 (33.3%) of the patients with familial IPF. Our results, which were established on an individual-patient level study design and in routine clinical practice (as opposed to the case-control study design), are roughly in line with the around 10% prevalence of causative TCG variants in patients with IPF.

## 1. Introduction

Short telomeres have long been connected to idiopathic interstitial pneumonias (IIPs); especially to the most common and most deadly of the IIPs, idiopathic pulmonary fibrosis (IPF) [1,2,3]. Not only in familial cases but short telomeres are frequently observed also in sporadic IPF patients (individuals with no family history of IIP) [1,3]. It is believed that telomere dysfunction, which results from (critically) short telomeres, triggers aberrant lung healing by fibroblasts, eventually leading to scar formation and pulmonary fibrosis [4]. Furthermore, in mouse models, telomere elongation (through activation of the telomerase enzyme) was shown to prevent the onset of lung profibrotic pathologies [5].

Telomere length, as measured in leukocytes, is determined by an array of environmental (exercise [6], bodyweight [7], smoking [8], etc.), demographic (age [9], gender [7]) and genetic [10,11] variables. Genetic variables with the highest impact on telomere length (shortness) are pathogenic-mostly ‘loss-of-function’-variants present in genes of the telomerase complex (telomerase complex genes (TCG)) [12].

At each cell replication, telomere length shortens for roughly 30 to 150 base pairs [13]. This progressive telomere shortening is due to the end replication problem of linear chromosomes and is offset by the action of the telomerase enzyme [13]. Therefore, pathogenic variants in TCGs which cause insufficiency of the enzyme lead to critically short telomeres sooner (after fewer cell replications) than if the insufficiency would not exist.

Pathogenic variants in TCGs cause a distinctive clinical phenotype (the so-called short telomere syndrome) which is characterized by (idiopathic) pulmonary fibrosis, bone marrow failure, liver cirrhosis and premature greying [14,15,16]. Due to genetic anticipation [15] and different levels of enzyme insufficiency caused by various pathogenic variants of the TCGs [17,18], the clinical presentation of short telomere syndrome can be diverse [15,16]; nevertheless, the most frequent clinical manifestation of short telomere syndrome is IPF [19].

Despite short telomeres being common in individuals with IPF [1,2,3], the causative variants in TCGs are not as often recognized. Before the widespread use of next generation sequencing (NGS), causative, pathogenic variants were discovered in up to 15% [20,21] of individuals with familial and in up to 3% [1,20] of individuals with sporadic IPF. However, after the dawn of the NGS, due to the wider availability of genetic testing and due to novel insights about the genetic etiology of IPF [18] these results have improved substantially. Rare, probably causative, variants in TCGs can now be identified in up to 25% [22,23] of individuals with familial and in about 10% [23,24] of individuals with sporadic IPF.

There are many reasons why the identification of causative TCG variants in patients with IPF is important from a clinical standpoint. Firstly, it offers the possibility of genetic counseling [22,25]. Secondly, it influences the prognosis [26,27] (i.e., after lung transplantation) and can have an impact on treatment decisions [28,29] (i.e., treatment adjustments after lung transplantation). Thirdly, due to genetic anticipation, an earlier and more severe onset of the disease manifests in successive generations, which also affects different organ systems [30], the offspring of individuals who inherit pathogenic variants in TCGs should be managed appropriately.

In light of the clinical implications of the genetic diagnosis, the recent (above mentioned) [23,24] results regarding the prevalence of TCG pathogenic variants in individuals with IPF are especially important. However, the design of the above studies was case-control at its core, meaning that the authors sought the enrichment of protein-altering, rare variants in TCG in cases compared to controls. While they found a statistically significant enrichment in cases, which likely points toward the important role of those variants in the disease pathogenesis, these results cannot be directly translated to routine practice since the studies did not provide variant classification [31] on an individual level. Such classification is of paramount importance when managing patients in routine practice. Therefore, to validate the above results regarding the prevalence of pathogenic TCG variants and to clarify the clinical utility of TCG variant testing in patients with IPF, we genotyped TCGs of a well-characterized cohort of patients with IPF for the presence of those variants.

## 2. Materials and Methods

### 2.1. Study Subjects

Twenty-seven consecutive patients that attended a routine check-up (between 1 January 2018, and 31 December 2018) due to management with anti-fibrotic therapy were included. In all, the diagnosis of IPF was previously (before the inclusion visit) established according to the ATS/ERS/JRS/ALAT guidelines [32]. As said, all patients were on anti-fibrotic therapy with either nintedanib or pirfenidone. The study was conducted in accordance with the amended Declaration of Helsinki. It was approved by the Slovenian National Medical Ethics Committee (approval number 0120-66/2021/3), and all patients gave their informed written consent.

### 2.2. Study Design and Sequencing

In all patients, NGS sequencing was performed with the following mostly telomere biology-related genes analyzed: *TERT*, *TERC*, *DKC1*, *TINF2*, *PARN*, *RTEL1*, *SFTPC*, *SFTPA2,* and *ABCA3*.

Next generation sequencing was performed on Illumina platforms and employed two schemes. The two schemes testing were used because in 2018 our laboratory routine genetic testing protocol for patients with IPF has switched from sequential testing to panel only testing. Therefore, in the first 12 patients (tested in early 2018) the sequential protocol was implemented with first performing Clinical Exome sequencing (TruSight One Panel (Illumina, San Diego, CA, USA)) and if no causative variant was identified the Whole Exome sequencing (SureSelect XT All Human exon (Agilent Technologies, Santa Clara, CA, USA)) ensued. In 15 patients (tested in late 2018) the panel-only protocol (QIAseq Targeted DNA Custom Panels (Qiagen, Hilden, Germany)) targeting nine genes mentioned above was used. We reached at least 50× median on target coverage in all cases, assuring a sufficient variant detection rate.

### 2.3. Data Analysis

The generated results were analyzed using a custom in-house pipeline based on the GATK best practices backbone. Alignment of reads to the human reference assembly (hg19) was performed using the Burrows-Wheeler (BWA) aligner [33], duplicate sequences removed using Picard MarkDuplicates [34], which was followed by base quality score recalibration, variant calling, variant quality score recalibration and variant filtering using elements of the GATK toolset [35]. Variant effect prediction was performed using ANNOVAR [36] and annotated using population-based annotation and pathogenicity prediction utilities (SIFT, Mutation Taster, PROVEAN, REVEL, MetaSVMP, FATHMM).

As already indicated, the analysis focused only on the nine aforementioned genes that are mostly telomere biology-related. Confirmatory Sanger sequencing was performed for all causative variants detected using the described NGS sequencing protocol.

## 3. Results

### 3.1. Clinical Characteristics

Demographic data, with age at diagnosis, current age, gender, family history of IIP, smoking status (only five individuals were never-smokers), pack-years smoked and IPF treatment are depicted in Table 1.

In three individuals, there was a family history of IIP. Patients in our cohort were slightly older at diagnosis (70.3 years vs. 67.3 years (*p* = 0.01 Welch’s t-test (26 degrees of freedom))), however, had the same gender distribution and smoking status history (Fisher’s exact test; *p* = 0.65 for gender and *p* = 0.10 for smoking status) as IPF patients in other cohorts (24).

### 3.2. Pathogenic Variants Identified in IPF Patients

According to the ACMG guidelines [31], pathogenic variants were found in 3 out of 27 (11.1%; 95% CI 2.4%–29.2%) patients; one had a family history of IPF. Hence, the genetic diagnosis was established in 3 out of 27 probands. Specifically, the pathogenic variants were detected in 2 out of 24 (8.3%; 95% CI 1.0%–27.0%) patients without a family history of IPF, classified as sporadic cases, and in 1 out of 3 (33.3%; 95% CI 0.8%–90.6%) patients with a family history of IPF.

Both variants identified in sporadic IPF cases were frameshift (null) variants, one located in the TERT gene (NM_198253.3: c.1374del) and the other in the RTEL1 gene (NM_001283009: c.326_329del). Both pathogenic variants were absent from the control population of the GnomAD project and were classified as pathogenic according to the ACMG guidelines [31] (Table 2).

In the patient with a family history of IPF (the proband has a son with IPF), we identified a missense likely pathogenic variant in the TERT gene. Identified missense variant NM_198253.3: c.368T > C was absent from the control population of the GnomAD project, and a deleterious effect on gene or gene product was uniformly predicted utilizing in silico predictors (SIFT, Mutation Taster, PROVEAN, REVEL, MetaSVMP, FATHMM). The same identified variant was present also in the affected son of the proband and the variant was, therefore, classified as likely pathogenic according to the ACMG guidelines [31] (Table 2).

Since only three individuals had a causative (pathogenic/likely-pathogenic) variant in the TCGs, we did not perform a formal statistical analysis comparing those patients to the rest of the group. However, 2 out of 3 patients with TCG causative variants were 61 years old at the time of diagnosis, and 61 years was the youngest age at diagnosis present in the whole cohort (only three individuals in total were 61 years old at diagnosis–all others were older). This fact points toward the idea that individuals with causative TCG variants are younger at disease onset than non-TCG patients with IPF [24].

## 4. Discussion

In the current study, we have evaluated the utility of pursuing a genetic diagnosis of TCG pathogenic variants in an unselected, well-characterized cohort of individuals with IPF. In all cohort patients, the diagnosis was established according to the ATS/ERS/JRS/ALAT guidelines [32], and all were on anti-fibrotic therapy with either nintedanib or pirfenidone. The genetic diagnosis, in accordance with the ACMG [31] criteria, was established in 3 out of 27 probands (11.1%); specifically in 1 out of 3 (33.3%) individuals with and in 2 out of 24 (8.3%;) individuals without a family history of IPF.

Albeit small, our study has found comparable results to other two contemporary and bigger studies that explored the prevalence of TCG causative variants in individuals with IPF [23,24]. However, there is a fundamental design difference between our and the two recent studies. While the two [23,24] sought for the enrichment of rare, protein-altering, qualifying variants on a populational level of individuals with IPF compared to controls (case-control design), we explored in how many individuals with IPF a pathogenic (likely pathogenic) variant according to the ACMG [31] criteria could be found. In routine clinical practice, it is important to follow the ACMG criteria, since only pathogenic/likely pathogenic variants can be used to inform clinical decision making (i.e., when used for prenatal genetic counseling).

In telomerase complex genes (at least in the *TERT* gene [23,24]), most rare protein-altering variants are missense in nature. A rare missense variant present in a gene known to cause a specific disease in an individual with that particular disease without a positive family history of the disease can most of the time be classified only as a variant of unknown significance (VUS) [31]. Therefore, in the above two studies, the missense variants (majority) in individuals with sporadic IPF (no family history of IPF) would, on an individual level, likely be classified only as VUS-implying that their role in the disease pathogenesis is unknown. However, in a case-control design, the enrichment of such variants (individual level VUS) in cases compared to controls points toward their significance in developing the disease.

In the Petrovski et al. study [23], the rare (minor allele frequency (MAF) < 0.05%), protein-altering, qualifying variant in TCG, was found in 13.6% of individuals with IPF. Specifically, it was found in 24.2% of individuals with familial (I)PF [23] and 11.3% of individuals with sporadic IPF. The prevalence in the Petrovski et al. study is slightly higher than the prevalence observed in our cohort. This could be due to the study design (IPF patients before lung transplantation), allowing for a slight enrichment of individuals with TCG pathogenic variants. Lung transplantation is usually offered to younger individuals, and individuals with TCG pathogenic variants tend to be younger at presentation than non-TCG IPF patients [24]. Indeed individuals in the Petrovski et al. study were younger than patients in usual IPF cohorts, including ours [22,23].

In the Dressen et al. study [24], roughly 9% of individuals with sporadic IPF carried a rare, protein-altering, qualifying variant in a TCG. The 9.31% prevalence was found when the MAF was set to <1%. In such a scenario, also 3.88% of individuals in the control population carried a ‘qualifying’ variant. However, when a more stringent MAF criterion, equal to the MAF level present in the Petrovski et al. study (MAF < 0.05%), which likely enriches for the true monogenic (high impact) variants, was applied, the frequency in cases (IPF patients) fell to 6.88%, however, the frequency in controls fell even more drastically to 1.61% [24]. The 1.61% frequency in controls is probably a mixture of some false positive variants (rare variants that do not have a role in disease pathogenesis) and true positive variants (rare variants with a role in disease pathogenesis) that reflect the natural course of the TCG mediated IPF. Here, the disease becomes clinically obvious when the telomere length falls below some critical value and is thus not only the result of the presence of the pathogenic variant but also of genetic anticipation. This latency period (time between the emergence of the pathogenic variant and clinical manifestation of the disease in a carrier of the variant) can be as long as 300 years and can span seven generations [17]. Therefore, a proportion of the 1.61% of controls with an ultra-rare (MAF < 0.05%) qualifying variant in TCGs probably represent case subjects in the latency period. Further supporting the theory is the fact that the majority (1.12%) of the 1.61% of control subjects with an ultra-rare qualifying TCG variant had a variant in the *RTEL1* gene [24]. Pathogenic variants in the *RTEL1* gene (together with pathogenic variants in the *PARN* gene) tend to have the lowest (of all TCG pathogenic variants) effect on telomere length [18], thus prolonging the latency period from the emergence of the variant to the clinically apparent disease onset.

According to the argumentation above, the proper frequency of pathogenic variants in currently known TCG in (sporadic) IPF lies somewhere in between ~6 and ~13% [23,24]. Our findings are in line with these results (~11% in the whole cohort and 8% in individuals with sporadic IPF).

According to some recommendations, individuals presenting with IPF who have a positive family history of IIP should be tested for the presence of germline pathogenic variants in TCGs [37]. However, around 10% of individuals with apparently sporadic IPF will subsequently have a bloodline relative diagnosed with an IIP during follow-up, sometimes many years later [37]. Therefore, the family-history selection criterion for choosing IPF patients eligible for genetic testing has drawbacks. A possible alternative in individuals with sporadic IPF is to measure their telomere length (TL), and in all IPF patients with TL below 10th age-standardized percentile (%), germline testing for TCG is performed. However, diagnostic TL testing with a recommended flow-cytometric technique [37] is cumbersome since it needs fresh blood to be sent to the certified laboratory that performs such measurements as time and sample sensitive procedures. Of note, our experience with the flow-cytometric methodology is somewhat disappointing since we sampled blood to measure telomere length four times (in two different patients) and got back an informative result only once. Furthermore, a proportion of TCG pathogenic variant carriers can have TL above the 10th age-standardized% [17,38]. Therefore, using a <10th age-standardized% TL based algorithm would miss those patients; however, knowing TCG variant status is important (as discussed in the introduction) for the management of patients with IPF as well as for the management of their family members.

In some disease settings, a 10% cut-off is established as the pre-test probability cut-off for having a causative genetic variant in order to test for that variant (i.e., the NICE guidelines recommend testing for a *BRCA1/2* variant status if a pre-test probability of an individual having a *BRCA* pathogenic variant is ≥10% [39]).

Considering (i.)the likely prevalence of TCG pathogenic variants among individuals with IPF (around 10%), (ii.) the possibility of “false negative” family history (in around 10% of patients), (iii.)the importance of obtaining a genetic diagnosis in patients with IPF, (iv.)the shortcomings of under the 10th age-standardized% TL diagnostic algorithm (cumbersomeness of flow-cytometry based testing and the possibility of missing mutation carriers), and (v.)the ever-growing ease/affordability of NGS genetic testing it might be reasonable that all individuals (regardless of family history) with IPF are offered genetic counseling. After the counseling session, the genetic counselor who weights all arguments for (i.e., presence of a family history of IIP, presence of other clinical signs of short telomere syndrome, etc.) and arguments against (higher age at presentation, heavy smoking history, etc.) genetic testing, decides whether to offer the testing to the patient.

## 5. Limitations/Strengths

The first and the most important limitation is the small sample size. The small sample size prevented us from conducting any formal statistical analysis and comparing our cohort to other IPF cohorts. Therefore, we were unable to make any statistically sound conclusions. Nevertheless, the prevalence of pathogenic TCG variants observed in our cohort was roughly similar to the prevalence found in larger studies [23,24]. Our study also has strengths. It was conducted on a prospective cohort of consecutive, well-characterized individuals with IPF, circumventing the inclusion bias and the bias connected to misdiagnosis. Furthermore, the study was conducted in a clinical setting, thus demonstrating the clinical utility of pursuing genetic diagnosis in individuals with IPF.

## 6. Conclusions

Taken together, the results of our study are roughly similar to the results of bigger studies [23,24] where around 10% prevalence of TCG pathogenic variants among individuals with IPF, regardless of the family history of the disease, was established. Due to the high proportion of the genetic causes of the disease, we, therefore, believe that all individuals with IPF are entitled to genetic counseling and that genetic counselors should have a low decision margin when offering genetic testing to such individuals.

## Figures and Tables

**Table 1:** Demographic and clinical data of IPF patients.

No.	Age at Diagnosis	Current Age ^b^	Sex	Family History of IIP	Smoking Status	Pack-Years	Treatment
1	65	69	F	Yes	Never smoker	/	Pirfenidone
**2 ^a^**	**61**	**68**	**M**	**No**	**Ex-smoker**	**27**	Nintedanib
3	67	deceased	M	No	Ex-smoker	10	Pirfenidone
4	64	70	F	No	Never smoker	/	Nintedanib
5	65	69	M	No	Ex-smoker	50	Nintedanib
6	70	deceased	F	No	Never smoker	/	Nintedanib
7	75	80	M	No	Ex-smoker	80	Pirfenidone
8	73	76	M	No	Ex-smoker	10	Pirfenidone
9	80	83	M	No	Ex-smoker	20	Nintedanib
10	79	82	M	No	Ex-smoker	20	Pirfenidone
**11 ^a^**	**68**	deceased	**M**	**No**	**Ex-smoker**	**30**	Pirfenidone
12	71	74	M	No	Ex-smoker	40	Nintedanib
13	75	78	M	No	Ex-smoker	50	Nintedanib
14	61	67	M	No	Ex-smoker	30	Nintedanib
15	75	79	M	No	Ex-smoker	8	Nintedanib
16	69	75	M	No	Ex-smoker	20	Nintedanib
17	67	70	M	No	Ex-smoker	20	Pirfenidone
18	72	deceased	M	No	Ex-smoker	10	Nintedanib
19	69	71	M	No	Ex-smoker	Unknown	Pirfenidone
20	69	76	M	No	Ex-smoker	20	Pirfenidone
21	72	75	M	No	Ex-smoker	Unknown	Pirfenidone
22	70	75	M	No	Ex-smoker	38	Pirfenidone
23	75	78	M	No	Ex-smoker	20	Pirfenidone
24	78	83	M	No	Ex-smoker	20	Pirfenidone
25	80	83	F	No	Never smoker	/	Pirfenidone
**26 ^a^**	**61**	deceased	**F**	**Yes**	**Never smoker**	/	**Pirfenidone**
27	67	69	M	Yes	Ex-smoker	25	Pirfenidone

Abbreviations: IIP, interstitial pneumonia; F, female; M, male; ^a^ Patients with identified causative (pathogenic/likely pathogenic) variants are in boldface. ^b^ deceased-individuals were dead at the time of article writing.

**Table 2:** Pathogenic variants found in our IPF patients.

Gene Symbol (Patient)	Transcript Change	Amino Acid Sequence Change	Variant Type	ACMG *
*RTEL1* *(pt-2)*	NM_001283009: c.326_329del	p.Ile110ThrfsTer40	Frameshift	Pathogenic
*TERT* *(pt-11)*	NM_198253.3: c.1374del	p.Trp459GlyfsTer50	Frameshift	Pathogenic
*TERT* *(pt-26)*	NM_198253.3: c.368T > C	p.Leu123Pro	Missense	Likely pathogenic

* Classification of variants according to the American College of Medical Genetics and Genomics (ACMG) guidelines [31].

## Data Availability

Data are contained within the article. Any needed clarifications and/or additional supporting data is available on request from the corresponding author (J.Š.).

## Abbreviations

ACMG	American College of Medical Genetics
IIP	Idiopathic interstitial pneumonia
IPF	Idiopathic pulmonary fibrosis
F	Female
M	Male
MAF	Minor allele frequency
NGS	Next generation sequencing
TCG	Telomerase complex genes
TL	Telomere length
VUS	Variant of unknown significance
